# Child transmission of SARS-CoV-2: a systematic review and meta-analysis

**DOI:** 10.1186/s12887-022-03175-8

**Published:** 2022-04-02

**Authors:** Sarah L Silverberg, Bei Yuan Zhang, Shu Nan Jessica Li, Conrad Burgert, Hennady P Shulha, Vanessa Kitchin, Laura Sauvé, Manish Sadarangani

**Affiliations:** 1 Department of Pediatrics, BC Children’s Hospital, 4500 Oak Street, V6H 3N1 Vancouver, BC Canada; 2 Faculty of Medicine, University of British Columbia, Vancouver, Canada; 3 Vaccine Evaluation Center, BC Children’s Hospital Research Institute, Vancouver, Canada; 4 BC Centre for Disease Control, Vancouver, Canada; 5 University of British Columbia Library, Vancouver, Canada

**Keywords:** COVID-19, Transmission, Pediatrics

## Abstract

**Background:**

Understanding of the role of children in COVID-19 transmission has significant implications for school and childcare policies, as well as appropriate targeting of vaccine campaigns. The objective of this systematic review was to identify the role of children in SARS-CoV-2 transmission to other children and adults.

**Methods:**

MEDLINE, EMBASE, CINAHL, Cochrane Central Register of Controlled Trials, and Web of Science were electronically searched for articles published before March 31, 2021. Studies of child-to-child and child-to-adult transmission and quantified the incidence of index and resulting secondary attack rates of children and adults in schools, households, and other congregate pediatric settings were identified. All articles describing confirmed transmission of SARS-CoV-2 from a child were included. PRISMA guidelines for data abstraction were followed, with each step conducted by two reviewers.

**Results:**

40 of 6110 articles identified met inclusion criteria. Overall, there were 0.8 secondary cases per primary index case, with a secondary attack rate of 8.4% among known contacts. The secondary attack rate was 26.4% among adult contacts versus 5.7% amongst child contacts. The pooled estimate of a contact of a pediatric index case being infected as secondary case was 0.10 (95% CI 0.03-0.25).

**Conclusions:**

Children transmit COVID-19 at a lower rate to children than to adults. Household adults are at highest risk of transmission from an infected child, more so than adults or children in other settings.

**Supplementary Information:**

The online version contains supplementary material available at 10.1186/s12887-022-03175-8.

## Background

The SARS-CoV-2 pandemic has led to worldwide economic disruption, as well as the mass-closing of social programs, daycare, and childcare institutions. Nearly 90% of students worldwide had their education disrupted by mid-April 2020 due to school closures [1]. To date, the data to support ongoing school closures to prevent increases in community SARS-CoV-2 transmission remain unclear. Some modeling studies have reported limited reduction in overall mortality rates of only 2-4% from school closures [2]. There have been reports of large-scale outbreaks associated with school openings [3], but this finding has not been consistently demonstrated in similar settings [4, 5]. In this context, it is critical to understand the role of children in transmission of the SARS-CoV-2 virus.

There has been greater focus on COVID-19 infection in adults, where the vast majority of symptomatic cases and deaths have occurred [1]. Adults are often index cases in household clusters due to their higher number of social contacts, and many studies focus on adult index cases in households. The prevalence of COVID-19 is much lower in children, as the incidence is consistently less than half that of adult cases [6, 7] In addition, children often present with asymptomatic or mildly symptomatic infection, and thus may be underrepresented in much of the literature [7, 8] There have been several studies synthesizing the state of current knowledge around pediatric presentation with COVID-19 or transmission to children from family members, but comparatively few studies that surround the transmissibility of pediatric infection [2, 9, 10] Those that have evaluate transmissibility systematically have largely done so early in the pandemic during maximal lockdowns and before many schools re-opened. This information can play an important role in decisions regarding reopening schools and the protective measures required in the classroom.

In this systematic review, we analyzed intra-familial and institutional spread of COVID-19 from a pediatric index case with confirmed child-to-adult or child-to-child infection. Our primary outcomes of interest were the secondary attack rates (SARs) of pediatric index cases in child and adult populations. We limited this review to contact-tracing studies with confirmed documented transmission to best characterize SARs.

## Methods

### Search strategy

For this systematic review and meta-analysis, we searched the literature to identify all published articles that reported evidence of SARS-CoV-2 transmission from children, either to other children or to adults. This study followed the Preferred Reporting Items for Systematic Reviews and Meta-Analyses (PRISMA) reporting guideline for meta-analyses (Supplementary Methods 1). We developed a search strategy to identify evidence in the literature of pediatric SARS-CoV-2 transmission and refined its parameters in consultation with a research librarian. We searched Ovid MEDLINE, EMBASE, CINAHL, Cochrane Central Register of Clinical Trials, and Web of Science databases for published studies in English exploring case-based pediatric COVID-19 transmission, published between January 1, 2020 to March 31, 2021 (Ovid MEDLINE search in Supplementary Methods 2).

### Inclusion/exclusion criteria

We included published articles in English that demonstrated likely or confirmed transmission of SARS-CoV-2 from a child to an adult and/or to another child, with children defined as 18 years of age or younger. Likely transmission was defined as probable, symptomatic cases without a confirmed nucleic acid test by polymerase chain reaction (PCR). Confirmed transmission was defined as secondary cases confirmed by PCR via nasopharyngeal or salivary sample or through local community testing practices. We excluded articles that only demonstrated adult-to-child transmission or that did not contain information regarding the transmission of SARS-CoV-2. We excluded articles commenting on (1) neonatal (<28 days old) occurrences of transmission, (2) antibody results rather than PCR confirmed SARS-CoV-2, and (3) transmission occurring in a hospital setting. Letters, editorials, pre-printed articles, and review articles containing no primary data were excluded. Inclusion and exclusion criteria were specified in advance and documented in the study protocol.

### Data extraction

**Fig. 1:**
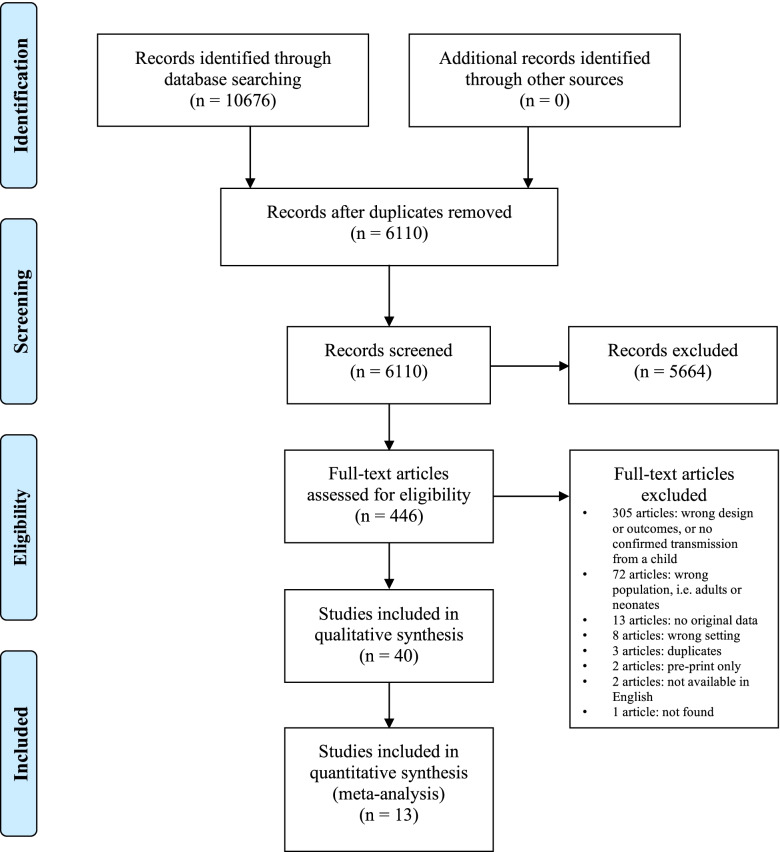
PRIMSA flow diagram

After eliminating duplicates, two reviewers (two of S.L.S., B.Y.Z., C.B., or S.N.J.L.) independently screened all titles and abstracts to identify potentially eligible studies (Fig. 1). Full-text studies were then reviewed by two authors for eligibility (two of S.L.S., B.Y.Z., C.B., or S.N.J.L.). Disagreements were resolved by group discussion and review by a third reviewer (one of S.L.S., B.Y.Z., C.B., or S.N.J.L.). Articles found to be possibly eligible were fully assessed against inclusion and exclusion criteria by two reviewers independently (two of S.L.S., B.Y.Z., C.B., or S.N.J.L.). We also hand searched cited references in all potentially eligible studies for additional studies and identified additional studies cited in relevant review articles.

The following data categories were collected when available: study design, country, patient demographics (age, sex, ethnicity), index case, transmission setting, and the number of exposed adults and children who were infected or uninfected.

### Statistical analysis

Secondary attack rates were defined as the proportion of confirmed infections among all contacts when the number of total contacts were known. Secondary cases were defined as the number of confirmed infections among all contacts when the total number of contacts was unspecified. Meta-analysis was performed to evaluate the difference between household and school transmission settings, as well as differences between child-to-child and child-to-adult transmission. Studies with counts less than 10 were excluded from the meta-analysis. Pooled odds ratios (ORs) or proportions and their 95% confidence intervals (CI) were calculated through a random effects model based on DerSimonian and Laird with 0.25 for continuity correction [11]. For comparison of transmission to child contacts versus adult contacts, analysis was done solely in the household (close contact) settings with OR as a measure of effect size. For each meta-analysis, heterogeneity across studies was evaluated using Cochrane’s Q test and the inconsistency index (*I*^2^). A Cochrane’s Q *P*-value <0.05 indicated significant heterogeneity and I^2^ with values above 50% suggested substantial heterogeneity [12]. Results are presented as forest plots with 95% CI. All statistical analyses were performed using RStudio, version 1.4.1106.

### Risk of bias assessment

Using the NIH Quality Assessment Tool for Case-Control Studies, two reviewers independently (two of S.L.S., B.Y.Z., C.B., or S.N.J.L.) rated the quality of included studies.

## Results

6110 studies were screened for eligibility; of those, 40 articles met the eligibility criteria and were included in qualitative and quantitative analysis (Table 1). No additional articles were added from review of references. The majority of studies identified cases solely using PCR, with 9 reporting additional serologies in some or all cases, one study using rapid tests in call cases, one study conducted viral cultures, and 4 conducting whole exome sequencing in some or all cases (Supplementary Table 1). Symptoms described amongst cases were in keeping with the relatively mild or asymptomatic infection described broadly in the literature (Supplementary Table 1).

**Table 1:** Characteristics of included studies

Article	Number of index patients	Child-to-child transmission			Child-to-adult transmission			Country	Setting
		Positive PCR	Symptomatic; unconfirmed PCR	Asymptomatic or negative PCR	Positive PCR	Symptomatic; unconfirmed PCR	Asymptomatic or negative PCR		
Posfay-Barbe et al. 2020	39	0	6	13	2	11	11	Switzerland	Household
Laws et al. 2021	12	1	0	15	2	0	17	USA	Household
Lopez et al. 2020	4	1	2	--	7	2	--	USA	Household
Macartney et al. 2020	12	2	--	196	1	--	101	Australia	Childcare
Yoon et al. 2021	1	0	1	153	0	1	35	South Korea	Childcare
Ehrhardt et al. 2020^a^	6	11	--	--	--	--	--	Germany	Childcare
Heavey et al. 2020	3	0	0	895	0	0	94	Ireland	School
Kim et al. 2021^b^	1	1	--	--	--	--	2	South Korea	Household
Drezner et al. 2020^c^	1	0	0	10	--	--	--	USA	Soccer
Gharekhanloo, Sedighi, and Khazaei 2020	1	1	0	0	0	0	0	Iran	Household
Wong et al. 2020^d^	1	0	0		1	0		South East Asia	School
Schwartz et al. 2020^e^	1	0	3	4	6	2	4	USA	Family gathering
Pray et al. 2020	1	1	6	5	--	--	--	USA	School Retreat
Fong et al. 2020	2	3	--	--	3	--	--	Hong Kong	Household
Pitman-Hunt et al. 2021^f^	2	1	--	--	0	--	--	USA	Household
Teherani et al. 2020	7	4	0	6	10	0	10	USA	Household
Okarska-Napierala, Mańdziuk, and Kuchar 2021	7	3	1	--	9	0	--	Poland	Nursery
Maltezou et al. 2020^g^	61	1	--	--	1	4	--	Greece	Household
Heudorf, Steul, and Gottschalk 2020	3	2	--	--	1	--	--	Germany	Household
Ji et al. 2020	2	1	--	--	4	--	--	China	Household
Lin et al. 2020^h^	2	0	0	1	1	0	3	China	Household
Yung et al. 2021	2	0	42	--	--	--	--	Singapore	School
Buonsenso, Danilo, and Graglia 2021	2	9	0	0	--	--	--	Italy	School
Cesilia et al. 2021	1	3	0	0	1	0	1	Indonesia	Household
Gillespie et al. 2021^i^	1	3	--	--	0	--	--	USA	Household and School
Shah, Kondre and Mavalankar 2021^j^	72	0	--	--	2	--	--	India	Household
Siegel et al. 2021^k^	1	12	--	--	2	--	--	USA	School
Brandal et al. 2021	13	2	--	--	1	--	--	Norway	School
Dawson et al. 2021^l^	1	1	--	--	0	--	--	USA	School
Lin et al. 2021	1	--	--	--	3	--	--	China	Household
Fiel-Ozores et al. 2021	1	--	--	--	1	--	--	Spain	Household
Gupta et al. 2021^m^	19	0	--	6	7	--	50	India	Household
Hershow et al. 2021^n^	40	4	--	--	1	--	--	USA	School
Gold et al. 2021^o^	1	2	--	--	--	--	--	USA	School
Soriano-Arandes et al. 2021	80	60	--	--	107	--	--	Spain	Household
Jordan et al. 2021	30	11	--	--	1	--	--	Spain	School
Abbas and Tornhage 2021	1	2	0	0	1	0	0	Sweden	Household
Lewis et al. 2021	1	1	--	--	2	--	--	USA	Household
Ismail et al. 2021	21	6	--	--	33	--	--	UK	School
Danis et al. 2020^p^	1	--	--	--	--	--	--	France	School

^a^: Transmission only reported on 6 of 137 index patients

^b^: Transmission only reported on 1 of 107 index patients

^c^: Transmission only reported on 1 of 2 index patients; other index patient self-isolated and had no contacts

^d^: total of 29 child and adult patients who were exposed but asymptomatic

^e^: index patient tested “negative” but likely testing error

^f^: reported household positive cases were excluded as pattern of transmission was not specified

^g^: concomitant COVID infections of 2 siblings but one tested first and was used as the index patient

^h^: Neonatal transmission was excluded. Child testing positive was asymptomatic.

^i^: School A was not included as index patient’s age was unknown

^j^: Study also reports 3 total positive secondary cases (age unknown) out of 278 total contacts

^k^: Total of 16 cases (unknown age) out of 320 contacts

^l^: Total of 1 case out of 102 tested contacts

^m^: Total of 9 cases (unknown age) out of 122 contacts

^n^: Data of tertiary transmission was excluded as secondary index patients were not specified

^o^: Other transmission clusters excluded as index patient was not specified

^p^: Total of 1 case (unknown age) out of 172 contact

Of the 40 articles included, 13 studies were conducted in the North America [13–25], 14 in Europe [4, 10, 26–37], 12 from Asia [38–49], and 1 from Australia [50]. Of the 40 articles, 23 identified household child-to-adult or child-to-child transmission [10, 13–15, 18, 19, 24–27, 29, 32–34, 38–42, 45–48]. Seventeen articles documented transmission at a school [4, 17–23, 28–30, 35–37, 43, 44, 50], 6 in a childcare setting, [19, 28, 29, 31, 49, 50] and 5 from other social gatherings [16, 17, 23, 24, 47] (Table 1). Studies evaluating transmission in school and daycare settings, as well as structured social gatherings such as extracurricular sports, document a variety of non-pharmacological interventions to limit viral spread, including mask wearing, physical distancing, and maintenance of limited group sizes without intermingling (Supplementary Table 1).

A total of 457 pediatric index cases were included amongst all settings, resulting in a total of 355 secondary infections, of which 149 were pediatric cases and 206 were adult cases (Table 2). Overall, there was a mean of 0.78 secondary cases per index case (Table 2).

**Table 2:** Secondary Case Rate by Study Setting

Setting	Number of Studies	Total Index Patients	Number of pediatric cases	Number of adult cases	Number of total cases	Case Rate^**a**^
**Childcare**	6	54	26	53	79	1.46
**Household**	22	314	85	159	244	0.78
**Social Event**	5	23	13	15	28	1.22
**School**	16	142	68	50	118	0.83
**All Settings**	39^b^	457	149	206	355	0.78

^a^Case rate defined as the number of confirmed infections among all contacts, not considering the total number of contacts as not all contacts were known across all studies.

^b^One study (Danis et al) was excluded because it did not differentiate between pediatric vs. adult cases

**Fig. 2:**
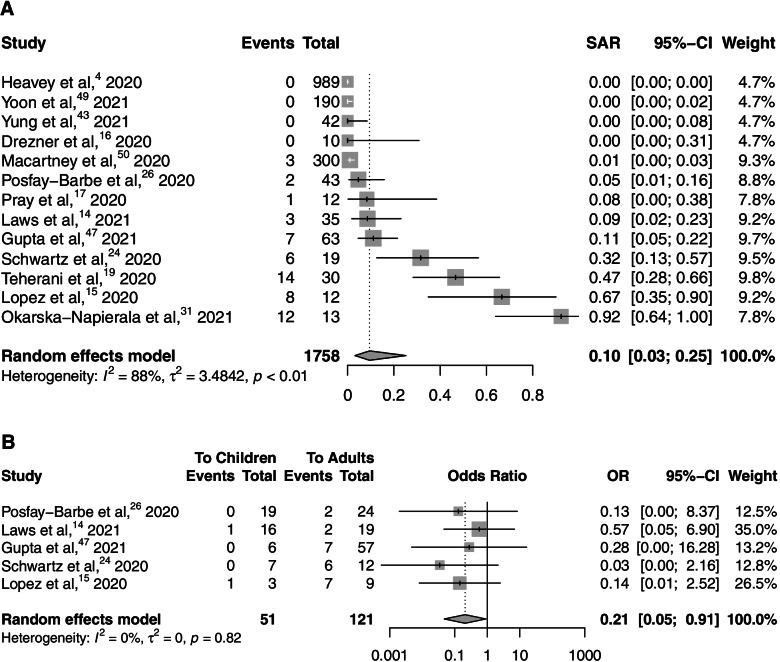
**A** Pooled estimates of secondary attack rate amongst child and adult contacts of pediatric index cases. **B** Pooled estimates of secondary attack rate amongst child versus adult contacts of pediatric index cases

In studies that documented the total contacts exposed, the child-to-child transmission rate was 5.7% while the child-to-adult transmission rate was 26.4%. Overall, in studies that documented the total contacts exposed, 8.4% of contacts exposed to a confirmed pediatric index case were infected. We undertook a meta-analysis of overall pooled SARs, with data included from 13 studies [4, 14–17, 19, 24, 26, 31, 43, 47, 49, 50]. The pooled estimate for all studies with full contact tracing of a contact of a pediatric index case being infected as secondary case was 0.10 (95% CI 0.03-0.25), with high heterogeneity (*I*^2^ = 88%) (Fig. 2).

We undertook a meta-analysis of pooled SARs in close contact settings to compare transmission to children and to adults, with data included from 5 studies [14, 15, 24, 26, 47]. The pooled OR estimate for adults was 0.21 (95% CI 0.05-0.91), with no heterogeneity (*I*^2^ = 0%) (Fig. 2B).

### Transmission Setting

There were 142 index patients documented in a school setting, with an overall mean of 0.83 secondary cases per index case (Table 3). In studies that documented the total contacts exposed, the child-to-child transmission rate in school settings was 2.0% of all child contacts, while the child-to-adult transmission rate in school settings was 11.7% (Table 3). Of all confirmed child-to-child transmission in school and childcare settings, almost half (48%) took place in secondary school environments.

**Table 3:** Secondary Attack Rate by Setting

	Childcare (6^**a**^)	Household (22^**b**^)	Social Event (5)	School (13^**c**^)	All Settings
**Child to Child Transmission (N)**	26/365	85/169	13/84	65/2304	149/2630
**Child to Child SAR (%)**	7.1%	50.3%	15.5%	2.0%	5.7%
**Child to Adult Transmission (N)**	53/167	159/338	15/73	50/426	208/789
**Child to Adult SAR (%)**	31.7%	47.0%	20.6%	11.7%	26.4%

^a^1 study excluded due to missing data

^b^10 studies excluded due to missing data

^c^6 studies excluded due to missing data

There were 314 index patients documented in a household setting, with an overall mean of 0.78 secondary cases per index case (Table 2). In studies that documented the total contacts exposed, the child-to-child transmission rate in household settings was 50.3% of all child contacts (Table 3). In studies that documented the total contacts exposed, the child-to-adult transmission rate in household settings was 47.0% of all adult contacts (Table 3).

**Fig. 3:**
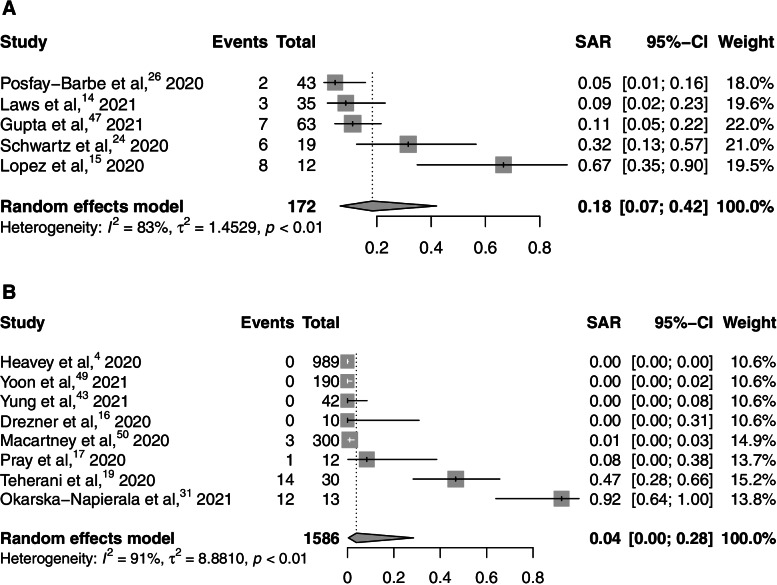
Pooled estimates of SAR amongst contacts of pediatric index cases in household and school settings

We undertook a meta-analysis of pooled SARs in household and school settings [4, 14–17, 19, 24, 26, 31, 43, 47, 49, 50]. The SAR estimate for household settings was 0.18 (95% CI 0.07-0.42), with significant heterogeneity (*I*^2^ = 83%), while the SAR estimate for school settings was 0.04 (95% CI 0.00-0.28) with significant heterogeneity (*I*^2^ = 91%) (Fig. 3). Comparisons of these pooled SARs by setting were insufficiently powered to run.

### Country of origin

Amongst European and North American studies, there was a mean of 0.98 and 0.84 secondary cases per index case respectively, while amongst Asian studies there was an overall lower rate of 0.30 secondary cases per index case (Table 4). In studies that documented the total contacts exposed, in North America, Europe, and Asia, the child-to-child transmission rate was 4.2%, 9.9%, and 4.4% while the child-to-adult transmission rate was 17.8%, 37.4%, and 21.4% respectively (Table 5).

**Table 4:** Secondary case rate by continent

Country	Number of Studies	Total Index Patients	Number of pediatric cases	Number of adult cases	Number of total cases	Case Rate
**All**	39^a^	457	149	206	355	0.78
**North America**	13	73	31	30	61	0.84
**Asia**	12	105	9	22	31	0.30
**Europe**	13	267	107	155	262	0.98
**Australia**	1	12	2	1	3	0.25

^a^One study (Danis) was excluded because it did not differentiate between pediatric vs. adult cases

**Table 5:** Secondary attack rates by continent

	North America (13^**a**^)	Asia (12^**b**^)	Europe (13^**c**^)	Australia (1)
**Child-to-Child Transmission (#)**	31/740	9/207	107/1085	2/198
**Child-to-Child Secondary Attack Rate (%)**	4.19%	4.35%	9.86%	1.0%
**Child-to-Adult Transmission (#)**	30/169	22/103	155/415	1/102
**Child-to-Adult Secondary Attack Rate (%)**	17.75%	21.4%	37.35%	1.0%

^a^4 studies excluded due to missing data

^b^5 studies excluded due to missing data

^c^6 studies excluded due to missing data

### Risk of bias assessment

Ten were deemed to be of good quality and have low risk of bias, while 22 were of fair quality and 8 were of poor quality (Supplementary Table 2). Studies deemed to be good quality all had clear study objectives, well-defined population groups, and consistently recruited from a single or homogeneous population. These studies had predefined exposure and outcome measures and defined timelines from exposure to outcome. Majority of these studies also had longitudinal follow-up to ensure delayed outcomes were adequately reported. Studies deemed to be fair or poor did not comment on potential confounding variables. Additionally, no studies reported the potential of multiple exposures or calculation of appropriate sample size.

## Discussion

To our knowledge, this is the most comprehensive systematic review of cases of pediatric COVID-19 transmission in the literature and is the first to capture data beyond the first global pandemic wave. Our review suggests that overall, children have posed a relatively small risk of transmission, particularly to other children, with an average of less than one secondary case per index case. There was limited evidence of transmission in the school or childcare setting, echoing reports from other closely studied school populations [50–52]. Our findings challenge many of the current public health practices of closing or limiting full time care for children in daycares, schools, and extra-curricular programming, particularly as most studies included in this review reflected periods with schools open for in class learning.

Although our study demonstrates that children do not appear to be a major contributor to the spread of COVID-19, adolescents may still play a role. The ages of all index patients and contacts were not available for meta-analysis. Of the children confirmed to have become infected with COVID-19 at school, almost half (48%) of them were confirmed to have been adolescents in a secondary school environment. This is likely to be an under-estimate as not all ages were specified. Further, the most significant reported school outbreaks took place in secondary schools [23, 28, 36]. Continuing extensive school closures in the setting of adult vaccinations and in the absence of an ongoing community outbreak, is unnecessary. Studies have increasingly shown such closures to be harmful to children, as the mental health effects and the additional unintended consequences to the most vulnerable children are coming to light [53–55]. As an increasing proportion of adults are vaccinated in populations, we must continue to monitor the role of children in COVID-19 disease burden and transmission because the role of children may change in the face of an increasingly immune adult population. This will enable evidence-based decisions on vaccination of children to be made, in light of anticipated data from pediatric clinical trials of COVID-19 vaccines and other key issues such as equitable global distribution of COVID-19 vaccines.

Our study demonstrated that the core population at risk of COVID-19 infection from a child are those residing in the same household as the child, with a secondary attack rate of 47% to adults and 50% of children in this setting. However, there have been numerous studies demonstrating that children are less likely to be the index case in households to begin with, and are less likely than other household members to be infected by a family member [56–59]. This may also be attributed to the more intimate caregiver roles that adults in the household may play compared to other siblings. Furthermore, while many public health policies recommend self-isolation within households when one individual is infected, this is usually unrealistic for children who rely on at least one caregiver for day-to-day needs. Alternatively, it is possible that the higher rate of transmission to adults in the household was a result of their biological vulnerability to the virus and population-wide increased rates of infection [60]. In studies that have sought to evaluate seroprevalence amongst household contacts, regardless of index case, there have been mixed findings, but overall children appear to have lower overall seropositivity than older household members [61–63]. This matches our findings of overall lower transmission to other children in the household compared to transmission to other household adults, and may again reflect differing susceptibility to the virus. While these studies and population-wide seroprevalence studies do not clearly demonstrate transmission risk and would therefore be unlikely to significantly change the interpretation of our review’s findings, their findings do correlate with the transmission dynamics we identified in this review.

There remains limited data available regarding the potential for pediatric COVID-19 transmission in larger pediatric group settings such as schools and summer camps. Only a few studies in these settings were included in our study, while other settings contained sufficient contact-tracing data to be fully included in this review [64, 65]. Our study demonstrates that if a child with COVID-19 does attend a congregate setting with other children, the relative risk to others is quite low. However, this does not preclude super-spreading events from occurring, particularly among adolescents. There have been reports of extensive and rapid transmission in settings with prolonged contact, even where some measures to mitigate introduction of infected participants into the setting [17]. The role of adults in these settings at assisting spread remains unclear, but necessary, as many reports from school and daycare settings report similar, if not higher, rates of spread from teachers themselves than from students [15, 66]. Close monitoring of congregate pediatric settings, particularly intimate ones such as summer camps, will be critical until mass vaccination is achieved.

Our review, completed through March 2021, represents transmission in the absence of vaccine pressure. Large-scale COVID-19 vaccination campaigns began after the timing of most studies included in the review. As well, most of the studies with available data took place prior to the widespread development of virus variants with different transmission data. The potential role of child-to-child transmission will likely become even more prominent as many countries are quickly achieving vaccination of significant proportions of their adult populations. Our study, which demonstrates low rates of child-to-child transmission, argues that in the context of widespread adult vaccination, re-opening of schools and other childcare settings will no longer be risky from a transmission standpoint and will be vital in face of the ongoing mental health and other widespread detriments of keeping such settings closed.

In this study, we were unable to exhaustively collect all evidence of children who were SARS-CoV-2 positive, and yet did not transmit the virus. These are the most reported pediatric cases of COVID-19 in the literature, particularly early in the epidemic when children had limited contacts outside of the home but could not be included as they provided no clear information regarding transmission. As a result, our review may over-report documented transmission from children. Additionally, due to the relatively mild or absent illness most children have with COVID-19, there is an unknown number of untested cases of SARS-CoV-2 amongst pediatric patients with unspecified transmission that we are unable to capture in this review. As a result, these asymptomatic scenarios may have caused us to under-estimate the true transmission rate. We sought to focus on symptomatic children who would be at highest risk of transmission to others, which may miss some transmission from asymptomatic cases. Like other contact tracing studies [56, 67], we were also unable to control for the possibility of a ‘common exposure’ where two individuals were infected by the same source at the same time, but only one of the two was identified as the index case. Furthermore, we were limited by the many reports of pediatric COVID-19 cases without contact tracing available, clarity on ages of all cases, and/or larger studies that report outbreak size in relation to a communal pediatric setting but did not undergo thorough contact tracing to document the nature of the spread. Reporting of secondary school cases was particularly limited by unspecified ages in multiple studies, likely leading to an under-representation of adolescent index cases in school settings. The bias introduced in this study, with studied individuals having potentially a more clearly defined exposure than those in population-wide studies, might therefore over-account for cases amongst close contacts. Lastly, as assessed by the NIH Quality Assessment Tool, the quality of included studies were mostly deemed fair. Specifically, domains that may affect the overall validity of our results come from the lack of reporting of potential multiple exposures and potential confounding variables. Consequently, without knowing other exposures or additional risk factors impacting transmissibility, our reported rates may have over-reported the true transmission rate. In summary, some factors lead to potential over-estimation of transmission compared to our findings, while others may contribute to potential under-estimation of transmission, but the relative contributions of these factors are difficult to ascertain.

Additionally, most studies did not report cycle threshold cut-off values for PCR testing for reporting positive results, limiting our interpretation of the infectious potential of studied cases amongst and between individual studies, although all studies followed national reporting guidelines in their individual jurisdictions. We also acknowledge that the transmission characteristics of new SARS-CoV-2 variants may differ. We did not identify studies that clearly reflected variants of concern circulating in the population at the time data was collected; a focus on the changing transmission dynamics, particularly in pediatric cases, will be critical for ongoing studies to monitor.

## Conclusions

In addition to the previously reports of children having milder disease course and better prognosis than adults, children also appear to be less likely to transmit COVID-19 than their adult counterparts. Household transmission remains the most prominent source of child-to-adult and child-to-child transmission. Further research is required to better understand how child transmission of COVID-19 has been impacted by the reopening of schools and the advancement of vaccines, as well as introduction of new variants.

## Supplementary Information

**Additional file 1: Supplementary Table 1. **Additional Study Characteristics.

**Additional file 2: Supplementary Table 2. **Risk of Bias Assessment.

**Additional file 3: Supplementary Methods 1. **PRISMA Checklist.

**Additional file 4: Supplementary Methods 2. **Database search.

## Data Availability

All data generated or analysed during this study are included in this published article [and its supplementary information files].

## Abbreviations

PRISMA: Preferred Reporting Items for Systematic Reviews and Meta-Analyses
PCR: polymerase chain reaction
OR: odds ratio
CI: confidence interval
SAR: secondary attack rate
