# Supplementary material for: Child transmission of SARS-CoV-2: a systematic review and meta-analysis
Source: BMC Pediatr. 2022 Apr 2;22:172. doi: 10.1186/s12887-022-03175-8 (PMC8975734; doi:10.1186/s12887-022-03175-8)
Supplement: Supplementary file 1 — Additional file 1: Supplementary Table 1. Additional Study Characteristics. [file 12887_2022_3175_MOESM1_ESM.docx]

**Supplementary table 1- online only: Additional study characteristics**

| **Study** | **Symptoms reported** | **Testing pattern and recruitment** | **Non-pharmacological interventions reported** | **Other reported testing** | **Cycle threshold and assay cut off** |
| --- | --- | --- | --- | --- | --- |
| Posfay-Barbe et al. 2020 | Amongst pediatric cases: cough, fever, nasal discharge, headache, sore throat, shortness of breath, myalgia, abdominal pain, arthralgia, anosmia, diarrhea, fatigue, rash, dysgueusia, nausea, vomiting, thoracic pain, conjunctivitis | All patients <16 years old with SARS-CoV- 2 infection were identified by means of the Geneva University Hospital’s surveillance network. Families called to follow up for contact tracing. Every patient with upper respiratory tract symptoms that presented to hospital was tested for SARS-CoV-2. | None specified. | None specified. | Not specified. |
| Laws et al. 2021 | Amongst pediatric cases: upper respiratory and neurologic symptoms were most common. Additional: headache, sore throat, rhinorrhea, nasal congestion, GI symptoms, cough, loss of smell, loss of taste, and fever. | Convenience sample of index patients with lab confirmed cases and all of their household contacts. | Report square footage per household and people per bathroom and bedroom | Used paired serologies for some but not all patients. | Not specified. |
| Lopez et al. 2020 | Not always reported; fever, fatigue, runny nose reported in some confirmed cases in Facility B. | Retrospective epidemiological contacts of childcare outbreaks in Salt Lake County, Utah, from April 1–July 10, 2020 | Not specified in all settings. Masks worn by childcare providers in Facility B. | None specified. | Not specified. |
| Macartney et al. 2020 | Not specified. | Close contacts - defined as children or staff with face-to- face contact for at least 15 min, or who shared a closed indoor space for at least 40 min with a case during their infectious period | From March 22, 2020, children were encouraged to stay home for distance learning until term 1 ended; however, schools remained open if home schooling was not an option. The follow-up period for close contacts of COVID-19 cases extended to May 1, 2020. All close contacts quarantined at home for 14 days. Schools and childcare settings closed temporarily on case notification and generally reopened within 24–48 h after environmental cleaning and public health measures instituted. | SARS-CoV-2-specific IgG, IgA, and IgM detection | Not specified. |
| Yoon et al. 2021 | Fever in child index case; fever; grandmother who he got it from was asymptomatic; cough myalgia in adult contact. | All children and staff members at the center were tested 8–9 days after the last exposure for SARS-CoV-2. Does not specify if all of family and other close contacts were tested. | Under the guidance of local health authorities, wearing masks, more frequent hand hygiene, and disinfection of the environment were required before the child tested positive. Adult staff at the center wore masks, but mask wearing by children were not consistent. | None specified. | Cycle threshold (Ct) value for envelope gene 19.0, positive cut-off value 40.0 |
| Ehrhardt et al. 2020 | Not specified. | Looked at all cases in the state, close contacts swabbed in schools, not specified whether home cases swabbed. | Schools open during the time examined. In schools and childcare: group sizes reduced by 50%, contact surfaces cleaned, regular and interim ventilation of rooms, exclusion of sick children, individual hygiene promotion, face masks outside classrooms in some jurisdictions but not all, physical distancing between children in secondary schools only, cancelation of wind instruments and singing in primary and secondary schools and some childcare facilities, and canceling of physical education in primary and secondary schools. | None specified. | Not specified. |
| Heavey et al. 2020 | Fever in 2/6, cough in 3/6, coryza in 1/6. | Contact tracing used but specifics not noted. | Before schools were closed. None otherwise specified. | None specified. | Not specified. |
| Kim et al. 2021 | Does not specify, just mentions ‘symptomatic.’ | All reported paediatric COVID-19 cases in South Korea with mass screening followed by comprehensive tracing of all identified contacts | If a confirmed paediatric case needs direct care by an uninfected guardian on hospitalisation, the guardian should wear a KF94 (N95 equivalent) mask, gloves, full body suit (or water- proof long-sleeve gowns) and goggles. All inbound international travellers were required to get tested and be quarantined at a government-designated facility (foreigners) or at home (nationals) for 14 days. All household contacts were screened with RT-PCR and were put under self-quarantine for 14 days, regardless of symptoms. | Assessed samples from nasopharynx, oropharynx, sputum, saliva, stool and plasma with PCR. | Ct value of ≤35 is positive and >40 is negative; value in between is reported as borderline. |
| Drezner et al. 2020 | Not specified. | All individuals with symptoms consistent with possible COVID-19 were encouraged to be tested | Teams divided into small groups of 5 or less. During the first 2 weeks, practices were limited to 1 team per soccer field with pods of up to 5 players divided into 4 field quadrants. After 2 weeks, 2 teams were allowed to practice on a field, with small group pods spaced into 8 field sections. Players were required to wear a mask to and from training, but use of a mask during exercise was optional. Players were always physically distanced from each other during training by a minimum of 6 feet at all times. Hand sanitizer encouraged before and after training. No group gathering before or after training. | None specified. | Not specified. |
| Gharekhanloo, Sedighi, and Khazaei 2020 | Fever, dry cough, and diarrhea; hypoxia requiring hospitalization | Not specified. | Not specified. | None specified. | Not specified. |
| Wong et al. 2020 | Mild cough | All school contacts via contact tracing | Not specified. | None specified. | Not specified. |
| Schwartz et al. 2020 | Nasal congestion | Investigation of outbreak by state health departments and CDC | Family did not distance or wear masks. Additional relatives only seen outside, maintaining physical distance. | Antibody testing conducted as well. | Not specified. |
| Pray et al. 2020 | Tabulated symptoms: some asymptomatic, others had shortness of breath, fever, cough, chills, sore throat, fatigue, myalgia, loss of taste or smell, diarrhea, nausea or vomiting, headache, congestion, or runny nose | All attendees at retreat offered testing. Contact tracing was done. | All attendees were required to provide documentation of either a positive serologic test result within the past 3 months or a negative RT-PCR tests result ≤7 days before travel, to self-quarantine within their households for 7 days before travel, and to wear masks during travel. At the retreat, students and counselors were not required to wear masks or social distance, and students mixed freely. Classes were held in outdoor pavilions with ~20 students per class seated <6 feet apart at tables. Teachers wore masks during class and were socially distanced from students at all times. The 127 students resided in dormitories (four to six per room) and yurts (eight per room), organized by grade. Beds in dormitory rooms and yurts were tightly spaced with three to four sets of bunks each, shared bathrooms, and shared common areas. Counselors roomed together in dormitories and yurts, and the four staff members resided in four separate housing units. Positive case was quarantined, and close contacts were then quarantined together in a separate dormitory. Students with mild symptoms given masks, but not isolated. | Serum IgG antibodies measured.  Whole genome sequencing done on some specimens (see next column) | Positive RT-PCR isolates with sufficient cycle threshold values were analysed with whole genome sequencing (on positive RT-PCR specimens with cycle threshold values less than 30) |
| Fong et al. 2020 | Not specified. | School-wide testing done for some but not all index cases. Close contacts surveyed. | Periods of school closure, with schools both open and closed during the time of assessment. Staff and students underwent daily temperature checks and wore face masks at all times. Schools switched from full days to half days, omitting lunch hours. Arrival and dismissal times were staggered using multiple entrances. Physical distancing in classrooms. Transparent partitions between desks at some schools. Limited group work and contact sports. Cancelled assemblies, extracurricular and after-school activities. | None specified. | Not specified. |
| Pitman-Hunt et al. 2021 | Fever, cough, sore throat, wheeze, chest pain, vomiting, diarrhea, and hypoxia noted in table | Contact tracing done retrospectively for positive children presenting to hospital and for following 6wks | Not specified. Detroit March-June 2020. | PCR assay or serum antibody testing. | Not specified. |
| Teherani et al. 2020 | Describes a number of contacts with symptoms, defined as “cough, shortness of breath (SOB), myalgias, loss of taste or smell *or* 2 of the fol- lowing symptoms: cough, myalgias, SOB, and/or loss of taste or smell” | Contact tracing done for children who tested positive in hospital | None specified. Atlanta March-June 2020. | None specified. | Not specified. |
| Okarska-Napierala, Mańdziuk, and Kuchar 2021 | Not specified. | After positive case noted, staff children and family members were all tested. | After lockdown was lifted. Children spent 8 hours there, divided into 3 groups, each cared for by 2 caregivers, no movement between groups. Caregivers wore masks. Parents did not enter the building when dropping off and picking up children. Contacts between parents and nursery workers lasted <15 minutes, with facemasks on. Family members of different children did not mix. | None specified. | Not specified. |
| Maltezou et al. 2020 | Tabulated symptoms: fever, low grade fever, runny nose, cough, headache, sore throat, diarrhea, loss of taste/smell, weakness, myalgia, dyspnea, nausea/vomiting, arthralgia, abdominal pain, restlessness/irritation | Identified through national registry – children with positive tests, families then contacted; as well pediatric hospitalizations in 9 hospitals tracked | School closure starting March 11, 2020. National lockdown March 23 – May 3. Lifting of lockdown May 4 – June 30. Schools reopened gradually depending on grade of students from May 11 – June 1, closed for summer on June 15. Students attended physically the schools every other day. | Viral load based on Ct values reported as low, medium or high (see next column). | Children were categorized into 3 groups based on the PCR amplification cycle threshold (Ct) value, as high, moderate, or low viral load (Ct <25, 25–30 or >30, respectively). Samples with Ct >38 were considered negative. |
| Heudorf, Steul, and Gottschalk 2020 | Symptoms not specified for children only; reports tabulated together with adults. Notes that 62% of children were asymptomatic, and 5% admitted to hospital. General symptoms reported include cough, fever, general symptoms, sniff, sore throat, diarrhea, loss of smell, loss of taste, hospitalisation, death. | All cases in Frankfurt tracked along with contact tracing details | Frankfurt March-July 2020. Children tested after possible contact in community facility, and extensive screening done for children in close contact with positive contact in any setting. In the State of Hesse, children’s facilities and schools were closed on March 16, 2020, with emergency care being offered for children of parents who work in the critical infrastructure. As of April 27, 2020, schools gradually reopened for the higher grades, obeying extensive preventive measures (small groups, distance 2 m, marked routes for in-school movement, lessons in person only every other day, with e-learning in between. As of May 18, all age groups, including the primary schools, returned to school and care facilities, adhering to strict hygiene requirements. Starting on June 22, regular operations during the COVID-19 pandemic took place with daily school lessons in person while still keeping to high hygiene standards; this was continued until the start of the school holidays on July 6, 2020. | None reported. “Genetic fingerprinting” to check whether the virus detected in adults and their children is identical was not carried out. | Not specified. |
| Ji et al. 2020 | Fever; asymptomatic; cough; acute respiratory illness | Contact tracing for confirmed positive children done | Transit in and out of city area stopped; significant screening done of symptomatic individuals; mandatory masks; voluntary quarantine; closure of factories, schools, businesses, and other institutions. In total, all of this done January – March 2020. | None specified. | Not specified. |
| Lin et al. 2020 | Nasal obstruction for 2 days, dry cough | Family evaluated. | Not specified. | None specified. | Not specified. |
| Yung et al. 2021 | Fever, cough, sore throat, rhinorrhea, shortness of breath, diarrhea noted in table amongst contacts. Some were noted to be asymptomatic. | Comprehensive surveillance and contact tracing of cases associated with seeding incidents in educational settings (identified from nationwide surveillance and contact tracing) | All close contacts placed in 14 day quarantine. Schools not routinely closed. Targeted public health measures including terminal cleaning of the schools and measures to reduce student mixing, such as suspension of extracurricular or sport activities and staggered recess breaks. Preschool was closed for 14 days after detection of increasing number of staff members positive. | None specified. | Not specified. |
| Buonsenso, Danilo, and Graglia 2021 | Pyrexia, headache, anosmia and ageusia. Previous contacts with ‘flu-like symptoms’ | Evaluation of classmates of a known case | Did not adhere to recommendations – class was not sub-divided, less than 1m between students, no hand sanitizer available. Windows were kept open. Students wore surgical mask in class. Adolescents requested to have lunch at own desks. | None specified. | Not specified. |
| Cesilia et al. 2021 | Student had pain while swallowing; hoarseness. | Evaluated a child returning from boarding school after a cluster was noted. | Not specified. | Rapid diagnostic test also used. | Not specified. |
| Gillespie et al. 2021 | Not specified. | Two schools monitored, specific population such as athletes tested more frequently. Tested entire school population after Thanksgiving break. | Each school followed the CDC’s guidelines designed to prevent the transmission of SARS-CoV-2 while also including aggressive laboratory screening similar to that which colleges and universities were implementing. Implemented with return to school in person. Extensive NPI reporting in article including maintaining 6ft spacing between students, enhanced filtration, masks at all times, cancelation of some activities, limiting talking during meal time. | Transitioned from nasal swabs to saliva samples for PCR. | Not specified. |
| Shah, Kondre and Mavalankar 2021 | Not specified. | 10% of all pediatric patients in Gujarat state selected and contact traced. | Not specified. | None specified. | Not specified. |
| Siegel et al. 2021 | Report just ‘symptomatic’ and refer to the CDC COVID-19 symptoms webpage. | Team assessed after high school football player tested positive; School environmental assessment then conducted | Mask use was infrequent during practice, and masks were not worn when playing other teams. Factors that likely contributed to team transmission included 1) infrequent mask use in the weight room or during practice; 2) inadequate physical distancing and air ventilation on buses transporting players (windows remained closed); 3) infrequent cleaning and disinfection of locker rooms, weight room equipment, and communal areas (e.g., hallways and bathrooms) before and after practices; and 4) insufficient sanitizing of shared hydration system drinking nozzles between uses. | None specified. | Not specified. |
| Brandal et al. 2021 | “Mild symptoms” and “asymptomatic” cases noted. | All confirmed pediatric cases in the studied counties during the observation period had all contacts tested twice during quarantine period | Schools followed national guidelines, which included strengthened hygiene measures, physical distancing and a clear message to stay home if symptomatic, even with mild symptoms. Use of face masks was not recommended in schools. | Saliva samples used for PCR. | Weakly positive noted as a Ct value of 35.73 in single patient. |
| Dawson et al. 2021 | Not specified. | School-based contacts of confirmed cases in studied schools in the county offered testing. | Modified quarantine policy permitting student close contacts aged ≤18 years who had school-associated contact with a person with COVID-19 and met masking requirements during their exposure to continue in-person learning. Close contacts of a person with COVID-19 were permitted to attend school in person during their quarantine period if 1) the school had a mask mandate, the school’s classrooms were arranged to maximize physical distancing, the school had increased hand hygiene practices, and the school screened students and staff members for COVID-19 symptoms and immediately isolated symptomatic persons and 2) the close contacts were K–12 students aged ≤18 years, their only exposure to the person with COVID-19 was in the educational environment (e.g., a classroom), they did not have prolonged (≥15 minutes) direct physical contact with the person with COVID-19, and the close contacts and person with COVID-19 had all been wearing masks appropriately during the time of exposure. Modifications to increase ventilation included opening windows or doors, fans, decreased occupancy, replacing or updating heating, ventilation, and air conditioning systems. | Whole genome sequencing (97%–99% coverage) was conducted on RT-PCR–positive saliva samples using Oxford Nanopore Technologies MinION sequencing at CDC. | Not specified. |
| Lin et al. 2021 | Fever, cough | Close contacts traced after an outbreak in Haikou, Hanan was noted. | Some contacts intermittently wore masks. | Serum IgG and IgM tested. Test result≥10.0 AU/mL was reported as positive. Throat swabs, sputum, feces, urine, breast milk, and other specimens collected and tested for SARS-CoV-2. | Not reported. RT-PCR method (Shanghai Geneo kit and/or Daan kit). Both open reading frames (ORF) and N sites were detected in the kits. A positive result was reported when both ORF and N sites were positive using the Shanghai Geneo Kit. Single site positives were either retested or resampled using Daan kit. Positive results were reported when the retested and/or resampled site was positive. |
| Fiel-Ozores et al. 2021 | Fever, headache, cough, asthenia, diarrhea, myalgia, breathing difficulty, cutaneous manifestations, odynophagia, chills, wheezing, anosmia/hyposmia, rhinorrhea, dysgeusia, abdominal pain, vomiting | All children in catchment who had a COVID-19 swab done for suspected symptoms over study time; interviewed patients and families. | Population-wide lockdown was in place during the study period. Schools remained open. | All patients included in the study also underwent testing for detection of anti-SARS-CoV-2 IgA and IgG antibodies | Not specified. Used STARlet handler (Micro- lab) and the STARMag 96 × 4 Universal Cartridge Kit. Nucleic acids were amplified and detected in a CFX-96 thermal cycler using the AllplexTM 2019-nCoV assay kit. |
| Gupta et al. 2021 | All asymptomatic | All asymptomatic travellers from states with a high incidence of COVID-19 were quarantined. Children tested first. Immediate family members of confirmed pediatric cases tested. | Quarantine period for inter-state travel. | None specified. | Not specified. |
| Hershow et al. 2021 | Some noted simply to be symptomatic, others asymptomatic. | Index cases were traced in schools after school reopening. School contacts were traced. school contact was defined as a student or staff member who was in contact with the index patient for a cumulative total of 15 minutes or more during a 24-hour period in a classroom, cafeteria, school bus, or recess space during an index patient’s infectious period. | Students were placed in cohorts by classroom whenever possible to reduce interactions between classes. Most schools staggered lunch, gym classes, and special activities, such as library use or art classes. At some schools, classes would mix by grade level at recess. Schools limited nonessential extracurricular in-person events, and other events (e.g., sports, assemblies, performances, and field trips) were held virtually when feasible. | Saliva used as alternate to NP swab.  Whole genome sequencing run on positive specimens. | Not specified. |
| Gold et al. 2021 | Some were symptomatic, but no further details provided. | Contact tracing of school-related clusters including household and school contacts. Testing offered to all contacts. | All nine transmission clusters involved less than ideal physical distancing, and five involved inadequate mask use. tudents and staff members exposed to a COVID-19 patient were advised to quarantine for a minimum of 7 days if a specimen collected ≥5 days after exposure was negative for SARS-CoV-2 and they remained asymptomatic or for 10 days if they were not tested and remained asymptomatic. Plastic dividers on desks were present but desks <3ft apart. The school district mandated in-classroom mask use except while eating. | None specified. | Not specified. |
| Soriano-Arandes et al. 2021 | Almost half were asymptomatic; others had fever, cough, headache, fatigue, diarrhea, abdominal pain, vomiting, anosmia, ageusia, skin lesions, dyspnea and some others. | Contact tracing done for each positive patient. | During summer break and after school re-opening. Nonpharmaceutical interventions were applied in all schools, including face masks in classrooms and school buildings in children older than 6 years. | None specified. | Not specified. |
| Jordan et al. 2021 | 50% of cases were asymptomatic. | Identified cases in 22 summer schools through surveillance program involving weekly saliva sampling during 2-5 weeks as well as )cases identified through the Catalonian Health Surveillance System. Contacts were then traced. | All centres followed prevention protocols: bubble groups of 8-14 children, frequent hand washing, facemasks mandatory for children older than 6 years, and recommended outside) and conducting activities mostly outdoors. | Saliva or NP swabs used for PCR.  Serology testing done at 0 and 5 weeks. | Not specified. |
| Abbas and Tornhage 2021 | One child had sore throat, headache, rhinorrhea, but was afebrile. Mother had fever, headache, fatigue. Another child had mild cold. Another child had fever, headache, fatigue, and later developed MIS-C symptoms requiring hospitalization. | All family members had PCR testing and later had serologies that were positive | Not specified. | Serologies were tested later on. | Used qualitative and quantitative assays. Qualitative cut off is 10AU/ml. Quantitative Cut-off Index S/C is 1.4. |
| Lewis et al. 2021 | Divided into classic, non-classic, and a-syndromic, but do not report symptoms by age (or index vs contacts) | Convenience sampling, chose 5 households | Not specified. | Viral cultures also collected | Specimens positive by RT- PCR that were collected on day 14 with Ct values <35 were also cultured. |
| Ismail et al. 2021 | Not specified. | Not specified. Per public health. Noted that some children tested because they were a household contact. | Schools had re-opened. Gradual easing of lockdown began on May 10 and, from June 1, included re-opening of some early years settings and primary and secondary school years following implementation of strict infection control measures, including smaller classes separated into distinct social bubbles that do not mix with other bubbles in the setting, physical distancing, and frequent handwashing. | None specified. | Not specified. |
| Danis et al. 2020 | Not all reported; index case not reported. | Both low and high risk contacts were tested | Per the French national guidance for contacts of Covid-19 cases, low risk contacts were asked to measure their body temperature twice a day during a 14-day period after their last exposure, and, in case of fever or respiratory symptoms to wear a surgical mask and contact the emergency hotline. In addition, high/moderate risk contacts were isolated at home, and were actively followed up through daily calls. Parents from school informed of contact; 2 schools closed for 2 weeks and 1 school closed for 1 week. Those in the same class as the contact were then isolated at home. | Viral load calculated. For 4 cases, a low viral load detected (often under the limit of quantification of 1 log10 copies/1000 cells). | Not specified. |
