# Supplementary material for: Child transmission of SARS-CoV-2: a systematic review and meta-analysis
Source: BMC Pediatr. 2022 Apr 2;22:172. doi: 10.1186/s12887-022-03175-8 (PMC8975734; doi:10.1186/s12887-022-03175-8)
Supplement: Supplementary file 2 — Additional file 2: Supplementary Table 2. Risk of Bias Assessment. [file 12887_2022_3175_MOESM2_ESM.docx]

Supplementary Table 2 – Online Only: Risk of bias assessment of all included studies according to the NIH Quality Assessment Tool for Observational Cohort and Cross-Sectional Studies

| **Study** | **1** | **2** | **3** | **4** | **5** | **6** | **7** | **8** | **9** | **10** | **11** | **12** | **13** | **14** | **Rating^a^** |
| --- | --- | --- | --- | --- | --- | --- | --- | --- | --- | --- | --- | --- | --- | --- | --- |
| Posfay-Barbe et al. 2020 | Yes | Yes | Yes | Yes | NR^b^ | CD^c^ | CD | NA^d^ | Yes | No | Yes | NA | NR | NR | Fair |
| Laws et al. 2021 | Yes | Yes | Yes | Yes | NR | Yes | Yes | NA | Yes | No | Yes | NA | NR | NR | Fair |
| Lopez et al. 2020 | Yes | Yes | Yes | Yes | NR | Yes | Yes | NA | Yes | No | Yes | NA | NR | NR | Fair |
| Macartney et al. 2020 | Yes | Yes | Yes | Yes | NR | Yes | Yes | Yes | Yes | No | Yes | NA | NR | NR | Good |
| Yoon et al. 2021 | Yes | Yes | Yes | Yes | NR | Yes | Yes | NA | Yes | No | Yes | NA | NR | NR | Fair |
| Ehrhardt et al. 2020 | Yes | Yes | Yes | Yes | NR | CD | CD | Yes | Yes | No | Yes | NA | NR | NR | Poor |
| Heavey et al. 2020 | Yes | Yes | Yes | Yes | NR | Yes | Yes | NA | Yes | No | Yes | NA | NR | NR | Fair |
| Kim et al. 2021^b^ | Yes | Yes | No | Yes | NR | Yes | Yes | NA | Yes | No | Yes | NA | NR | NR | Fair |
| Drezner et al. 2020 | Yes | Yes | Yes | Yes | NR | CD | CD | NA | Yes | No | Yes | NA | NR | NR | Fair |
| Gharekhanloo, Sedighi, and Khazaei 2020 | Yes | No | No | Yes | NR | CD | NA | NA | Yes | No | NA | NA | NA | NA | Poor |
| Wong et al. 2020 | No | Yes | Yes | Yes | NR | Yes | Yes | NA | Yes | No | Yes | NA | NR | NR | Fair |
| Schwartz et al. 2020 | Yes | No | No | Yes | NR | Yes | Yes | Yes | Yes | No | Yes | NA | NR | NR | Poor |
| Pray et al. 2020 | No | No | No | Yes | NR | Yes | Yes | NA | Yes | No | Yes | NA | NR | NR | Fair |
| Fong et al. 2020 | No | Yes | Yes | Yes | NR | Yes | Yes | NA | Yes | No | Yes | NA | NR | NR | Fair |
| Pitman-Hunt et al. 2021 | Yes | No | Yes | Yes | NR | CD | CD | NA | Yes | No | Yes | NA | NR | NR | Fair |
| Teherani et al. 2020 | Yes | Yes | Yes | Yes | NR | CD | CD | Yes | Yes | No | Yes | NA | NR | NR | Fair |
| Okarska-Napierala, Mańdziuk, and Kuchar 2021 | No | No | No | Yes | NR | Yes | Yes | NA | Yes | No | Yes | NA | NR | NR | Fair |
| Maltezou et al. 2020 | Yes | Yes | Yes | Yes | NR | CD | CD | NA | Yes | No | Yes | NA | NR | NR | Good |
| Heudorf, Steul, and Gottschalk 2020 | Yes | Yes | No | Yes | NR | CD | CD | NA | Yes | No | Yes | NA | NR | NR | Fair |
| Ji et al. 2020 | Yes | Yes | Yes | Yes | NR | Yes | Yes | NA | Yes | No | Yes | NA | NR | NR | Poor |
| Lin et al. 2020 | Yes | No | No | Yes | NR | Yes | Yes | NA | Yes | No | Yes | NA | NR | No | Poor |
| Yung et al. 2021 | Yes | Yes | Yes | Yes | NR | CD | CD | NA | Yes | No | Yes | NA | NR | No | Fair |
| Buonsenso, Danilo, and Graglia 2021 | Yes | No | Yes | Yes | NR | CD | CD | NA | Yes | No | Yes | NA | NR | No | Poor |
| Cesilia et al. 2021 | No | No | Yes | Yes | NR | Yes | NA | NA | Yes | No | NA | NA | NA | NA | Fair |
| Gillespie et al. 2021 | Yes | No | Yes | Yes | NR | Yes | Yes | Yes | Yes | No | Yes | NA | NR | NR | Good |
| Shah, Kondre and Mavalankar 2021 | Yes | Yes | Yes | Yes | NR | CD | CD | NA | Yes | No | Yes | NA | NR | NR | Good |
| Siegel et al. 2021 | No | No | Yes | Yes | NR | Yes | Yes | Yes | Yes | No | Yes | NA | NR | NR | Poor |
| Brandal et al. 2021 | Yes | Yes | Yes | Yes | NR | Yes | Yes | NA | Yes | No | Yes | NA | NR | NR | Good |
| **Study** | **1** | **2** | **3** | **4** | **5** | **6** | **7** | **8** | **9** | **10** | **11** | **12** | **13** | **14** | **Rating^a^** |
| Dawson et al. 2021 | Yes | Yes | Yes | Yes | NR | CD | CD | NA | Yes | No | Yes | NA | NR | NR | Fair |
| Lin et al. 2021 | Yes | No | Yes | Yes | NR | Yes | Yes | NA | Yes | No | Yes | NA | NR | NR | Fair |
| Fiel-Ozores et al. 2021 | Yes | Yes | Yes | Yes | NR | CD | CD | NA | Yes | No | Yes | NA | NR | NR | Poor |
| Gupta et al. 2021 | Yes | Yes | Yes | Yes | NR | Yes | Yes | Yes | Yes | No | Yes | NA | NR | NR | Fair |
| Hershow et al. 2021 | Yes | Yes | Yes | Yes | NR | Yes | Yes | NA | Yes | No | Yes | NA | NR | NR | Good |
| Gold et al. 2021 | Yes | Yes | Yes | Yes | NR | CD | CD | NA | Yes | No | Yes | NA | NR | NR | Good |
| Soriano-Arandes et al. 2021 | Yes | Yes | Yes | Yes | NR | CD | CD | NA | Yes | No | Yes | NA | NR | NR | Good |
| Jordan et al. 2021 | Yes | Yes | Yes | Yes | NR | Yes | Yes | NA | Yes | No | Yes | NA | NR | NR | Good |
| Abbas and Tornhage 2021 | Yes | No | Yes | Yes | NR | Yes | Yes | NA | Yes | No | Yes | NA | NR | NR | Fair |
| Lewis et al. 2021 | Yes | Yes | Yes | Yes | NR | Yes | Yes | NA | Yes | No | Yes | NA | NR | NR | Fair |
| Ismail et al. 2021 | Yes | Yes | Yes | Yes | NR | Yes | Yes | Yes | Yes | No | Yes | NA | NR | NR | Good |
| Danis et al. 2020 | No | No | Yes | Yes | NR | CD | CD | NA | CD | No | Yes | NA | NR | NR | Fair |

^a^The NIH Quality Assessment Tool for Observational Cohort and Cross-Sectional Studies (NIH) poses 14 questions: 1 = Was the study question or objectives clearly stated?; 2 = Was the study population clearly specified and defined?; 3 = Was the participation rate of eligible persons at least 50%?; 4 = Were all the subjects selected or recruited from the same or similar populations?; 5 = Was a sample size justification, power description, or variance and effect estimates provided?; 6 = For the analyses in this paper, were the exposures of interest measured prior to the outcomes being measured?; 7 = Was the timeframe sufficient so that one could reasonably expect to see an association between exposure and outcome if it existed?; 8 = For exposures that can vary in amount of level, did the study examine different levels of exposure as related to the outcome?; 9 = Were the exposure measures clearly defined, valid, reliable, and implemented consistently across all study participants?; 10 = Was the exposure assessed more than once over time?; 11 = Were the outcome measures clearly defined, valid, reliable, and implemented consistently across all study participants?; 12 = Were the outcome assessors blinded to the exposure status of participants?; 13 = Was loss to follow-up after baseline 20% or less?; 14 = Were key potential confounding variables measured and adjusted statistically for their impact on the relationship between exposures and outcomes?

^b^NR = Not Reported

^c^CD = Cannot Determine

^d^NA = Not Applicable
