# Supplementary material for: Child transmission of SARS-CoV-2: a systematic review and meta-analysis
Source: BMC Pediatr. 2022 Apr 2;22:172. doi: 10.1186/s12887-022-03175-8 (PMC8975734; doi:10.1186/s12887-022-03175-8)
Supplement: Supplementary file 4 — Additional file 4: Supplementary Methods 2. Database search. [file 12887_2022_3175_MOESM4_ESM.docx]

Methods 2 – Online Only: Database search

Database: MEDLINE

Ran: March 31, 2021

1. ((((exp Coronavirus/ or exp Coronavirus Infections/ or (D614G or coronavirus* or corona virus* or OC43 or NL63 or 229E or HKU1 or HCoV* or ncov* or covid* or sars-cov* or sarscov* or Sars-coronavirus* or Severe Acute Respiratory Syndrome Coronavirus*).mp.) and ((20191* or 202*).dp. or 20190101:20301231.(ep).)) not (SARS or SARS-CoV or MERS or MERS-CoV or Middle East respiratory syndrome or camel* or dromedar* or equine or coronary or coronal or covidence* or covidien or influenza virus or HIV or bovine or calves or TGEV or feline or porcine or BCoV or PED or PEDV or PDCoV or FIPV or FCoV or SADS-CoV or canine or CCov or zoonotic or avian influenza or H1N1 or H5N1 or H5N6 or IBV or murine corona*).mp.) or ((((pneumonia or covid* or coronavirus* or corona virus* or ncov* or 2019-ncov or sars*).mp. or exp pneumonia/) and Wuhan.mp.) or (2019-ncov or ncov19 or ncov-19 or 2019-novel CoV or sars-cov2 or sars-cov-2 or sarscov2 or sarscov-2 or Sars-coronavirus2 or Sars-coronavirus-2 or SARS-like coronavirus* or coronavirus-19 or covid19 or covid-19 or covid 2019 or ((novel or new or nouveau) adj2 (CoV on nCoV or covid or coronavirus* or corona virus or Pandemi*2)) or ((covid or covid19 or covid-19) and pandemic*2) or (coronavirus* and pneumonia)).mp. or COVID-19.rx,px,ox. or severe acute respiratory syndrome coronavirus 2.os.)) and 20191201:20301231.(dt).

2. Pediatrics/

3. Adolescent/

4. exp Child/

5. Infant/

6. (pediatric* or paediatric* or child* or youth* or adolescent* or juvenile* or teen* or infan* or newborn or baby or babies or neonat* or preschool* or pre-school* or kindergarten* or kindergarden* or elementary school* or nursery* or (day care* not adult*) or schoolchild* or toddler* or boy or boys or girl* or middle school* or high school* or pre-term or preterm* or premature birth* or NICU).mp. [mp=title, abstract, original title, name of substance word, subject heading word, floating sub-heading word, keyword heading word, organism supplementary concept word, protocol supplementary concept word, rare disease supplementary concept word, unique identifier, synonyms]

7. 2 or 3 or 4 or 5 or 6

8. Pneumonia, Viral/tm [Transmission]

9. Infectious Disease Transmission, Patient-to-Professional/ or Disease Transmission, Infectious/ or Infectious Disease Transmission, Vertical/ or Infectious Disease Transmission, Professional-to-Patient/

10. (transmi* or index or spread* or catch* or caught or carry*).mp. [mp=title, abstract, original title, name of substance word, subject heading word, floating sub-heading word, keyword heading word, organism supplementary concept word, protocol supplementary concept word, rare disease supplementary concept word, unique identifier, synonyms]

11. ((aerosol* or cough* or droplet* or infection* or infectious or disease*) adj3 (generat* or induc* or stimulat* or produc*or creat* or respirable range* or dispers* or transmission or transmitted or transmit or spread* or disseminat* or count* or precaution* or control* or inhibit* or prevent* or reduc*)).mp. [mp=title, abstract, original title, name of substance word, subject heading word, floating sub-heading word, keyword heading word, organism supplementary concept word, protocol supplementary concept word, rare disease supplementary concept word, unique identifier, synonyms]

12. 8 or 9 or 10

13. 1 and 7 and 12

14. 8 or 9 or 10 or 11

15. 1 and 7 and 14

16. limit 15 to english language
